# Seasonal variation in accelerometer-determined sedentary behaviour and physical activity in children: a review

**DOI:** 10.1186/1479-5868-9-49

**Published:** 2012-04-30

**Authors:** Carly Rich, Lucy J Griffiths, Carol Dezateux

**Affiliations:** 1 MRC Centre of Epidemiology for Child Health, UCL Institute of Child Health, 30 Guilford Street, London, UK, WC1N 1EH

**Keywords:** Accelerometer, Season, Child, Physical activity, Sedentary Behaviour, Review

## Abstract

**Aim:**

To undertake a review of the methods and findings of published research evaluating the influence of season on accelerometer-determined sedentary behaviour (SB) and physical activity (PA) in children.

**Methods:**

A literature search was carried out using PubMed, Embase, Medline and Web of Science up to, and including, June 2011. The search strategy focused on four key elements: children, SB or PA, season and accelerometer. Articles were eligible for inclusion if they were published in English, included healthy study participants aged ≤ 18 years, reported at least one outcome variable derived from accelerometer-determined measurements, and compared SB or PA between two or more seasons, or controlled for season of measurement. Eligible papers were reviewed and evidence tables compiled reporting on publication year, country studied, study recruitment, consent rate, sample descriptives, study design, accelerometer protocol, valid accelerometer data receipt, season definition, statistical methods and key findings.

**Results:**

Sixteen of 819 articles were eligible for inclusion: children aged two to five years, six to twelve, or six to 18 years were included in five, six and five articles respectively. Six articles were from the UK, six from other European countries, three from the USA and one from New Zealand. Study sample sizes ranged from 64 to 5595. PA was reported in all articles but SB in only three. Only four studies were longitudinal and none of these reported SB. Seasonal variation in PA was reported in all UK studies, being highest in summer and lowest in winter. In four non-UK studies seasonal variation in PA was not found. Findings were inconclusive for SB.

**Conclusion:**

There is sufficient evidence to support public health interventions aimed at increasing PA during winter in UK children. No conclusions can be drawn regarding the effect of season on children’s SB reflecting few studies of small sample size, lack of repeat measures, incomparable definitions of season and inconsistent accelerometer protocols. Future research should determine factors that drive seasonal patterns in PA and SB in children such as age, sex, and geographic and climatic setting to inform interventions and target populations.

## Introduction

Accelerometer-based measures of physical activity (PA) and sedentary behaviour (SB) are being increasingly obtained in large-scale studies to determine the level and pattern of children’s PA and SB, their determinants and relation to health outcomes. Such studies can help to identify optimal patterns of activity associated with future health and well-being and inform interventions to help populations meet these optimal patterns. This is essential as there is increasing evidence that PA patterns are established in childhood [1], that maintenance of an active lifestyle in adult life is protective against many chronic diseases [2-4], and that - in children as well as in adults - levels of PA have declined with successive generations in developed countries and those in economic transition [5].

A season is a division of the year marked by changes in weather, ecology and hours of daylight which have the potential to influence PA and SB. Periods of low temperatures, high rain fall, strong winds and snow may reduce the likelihood of children being physically active. Although the meteorological factors associated with seasons cannot be changed, the ability to identify specific seasons that are characterised by low PA levels and/or high periods of SB is important for the design of future public health interventions aimed at promoting PA and reducing SB.

It is also important to account for seasonal influence on PA and SB as large-scale cross-sectional studies rely on measurements made in different individuals and in different seasons, potentially introducing bias in between-subject differences in the assessment of habitual activity levels.

While the influence of season on PA levels has been reviewed by others these have, for the most part, focussed on adults [6-8] rather than children, and the extent to which findings can be reliably extrapolated to children is uncertain. Furthermore, the only review to concentrate on children [9] did not focus on accelerometer-determined activity.

In recent years accelerometers have been regarded as the ‘gold standard’ method to examine PA in childhood populations [10,11], as reliance on self or parent proxy reports may overestimate PA levels [12]. Carson *et al.*[9], summarising data from 35 studies in children, reported that in the majority (83%) a seasonal variation in children’s PA was found. However, studies based on accelerometer-determined activity were not reported separately, a number of major studies [13-19] published on this topic were omitted and the influence of seasonality on SB was not examined. As SB is not simply the absence of PA, but involves purposeful engagement in activities that involve minimal movement and low energy expenditure [20], seasonality may exert a different influence on SB than on PA in children.

We aimed to update and extend the review published by Carson *et al.*[9] by reviewing the influence of season on accelerometer-determined measures of PA and SB in children including studies published up to and including June 2011. The main purpose is to describe the current studies and their population coverage, critically appraise the study design and analytic methods used, and identify any significant gaps in evidence to inform public health guidelines and policy on optimal PA and SB levels in children.

## Methods

### Search strategy

We searched Embase, Medline, PubMed, and the Web of Science electronic databases (see search strategy illustrated in Additional file 1). This search strategy focused on four key elements: children, PA or SB, season, and accelerometer. Further studies were identified by hand-searching the bibliographies of published reviews and all included studies.

### Inclusion criteria

Studies were included if a full article was available, published in English, and included in the database from the year of inception up to and including June 2011 when the final searches were run. Any study or methodological design was included provided at least one outcome variable of PA or SB derived from accelerometer-determined measurements was reported, PA or SB were compared between at least two seasons, or adjustment was made for season of measurement. Articles were included if study participants, or a clearly defined subgroup, comprised children aged two to 18 years that were not selected on the basis of having a specific disease or health problem. Articles were not restricted according to study sample size or country of origin.

### Data extraction and analysis

Eligible papers were reviewed and evidence tables compiled reporting on year of publication, country studied, recruitment procedure, consent rate, sample size, age range, sex distribution, study design (cross sectional vs. longitudinal), valid accelerometer data receipt (children providing accelerometer data meeting individual study definition of ‘valid data’), definition of season employed, accelerometer protocol and outcome variables reported, statistical methods and key findings. The NHS Centre for Reviews and Dissemination [21] proposes a simple assessment to guarantee a minimum level of quality based on study design. However, a preliminary review of abstracts suggested that nearly all of the studies included in this review were observational studies and therefore poorly differentiated by this study hierarchy. Nevertheless, information relevant to the methodological quality of each article including the recruitment and sampling procedures, bias in consent and valid accelerometer data receipt are reported. The results of the review are presented as a qualitative review; no attempt has been made to synthesise statistical outcomes as the lack of uniformity across research methodology was too great to permit this.

### Searches and abstract review

**Figure 1:**
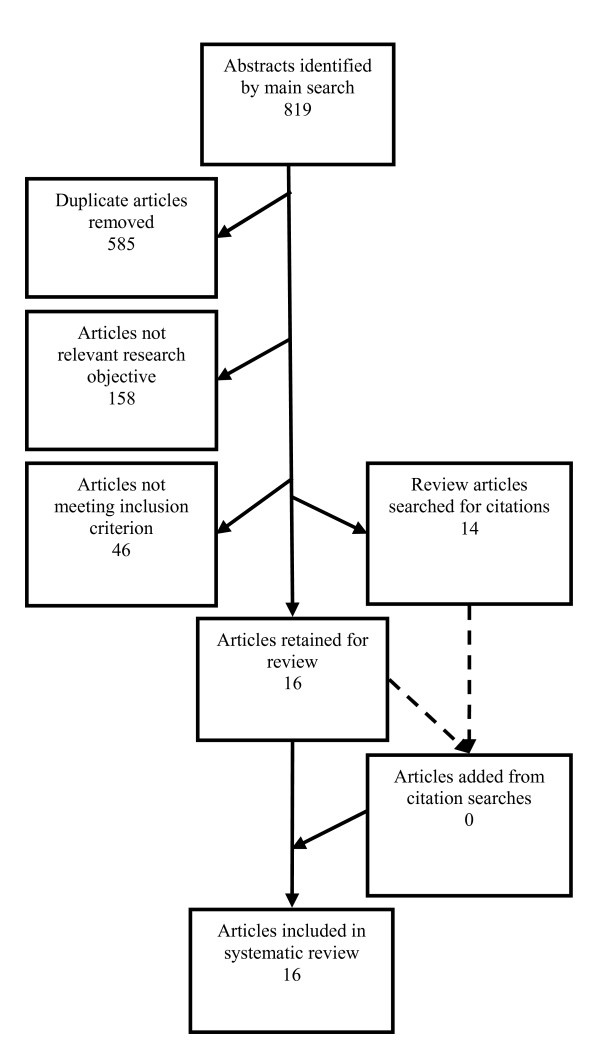
**Flowchart of articles (*n*** **= 16) included in review.**

**Figure 2:**
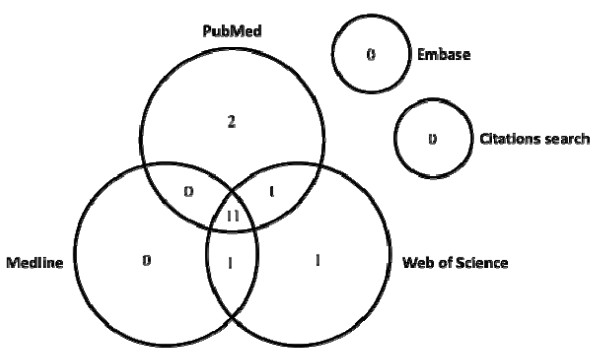
**Articles (*n*** **= 16) included in the review identified by each electronic database.**

A total of 819 abstracts were identified by the final electronic searches (219 in PubMed, 157 in Embase, 134 in Medline and 309 in Web of Science), of which 585 were duplicates, leaving 234 unique articles (Figure 1). Of these, 158 were not relevant to the research objective and 46 did not meet inclusion criteria, leaving 14 review and 16 original research articles. No further articles were identified by hand-searching of bibliographies, leaving 16 articles to be included in the review. Of these 11 were found in three databases, two in two and three in one only. No articles were identified through Embase (Figure 2).

## Results

### Overview of studies

Sixteen articles (Additional file 2: Table S1) were identified that reported 13 different studies assessing seasonal variation in accelerometer-determined PA in children, and in three this included SB. The dates of publication ranged from 2002 [22] to 2010 [14] and date of data collection from 1997 [16,23] to 2007 [14,18,24,25]. Twelve authors reported studies from Europe [13-18,23,24,26-29] (including six from the UK), three from the USA [19,22,30], and one from New Zealand [25]. In 12 studies between-subject seasonal differences in PA were assessed [14-16,18,19,22,23,25,26,28-30], and in the remaining four within-subject differences were evaluated through repeated accelerometer measurements in the same individuals (referred to here as longitudinal studies) [13,17,24,27].

### Sample characteristics

The sample sizes for studies that used a repeated measures design ranged between 64 [24] and 315 [27], and between 209 [26] and 5595 [28] for studies examining seasonal differences between subjects. Children aged two to five years, six to 12 years, or six to 18 years were included in five [19,22,25,26,30], six [14,17,18,24,27,28] and five [13,15,16,23,29] studies respectively. In 13 articles a measure of weight status was reported [14-17,19,22-29] while in only four was ethnicity [18,19,22,30] or socioeconomic status [13,14,22,23] of the sample reported.

### Accelerometer measurement protocol

The Actigraph was the most commonly used accelerometer by studies included within this review and there is extensive literature on its use to measure PA in children supporting its reproducibility, validity and feasibility in large samples of children [31]. In nine studies [13-16,23,26-29] the Actigraph 7164 were used, in two the Actigraph GT1M [18,24], in three the Actiwatch [17,19,22], with one study each for the Tritac R3D accelerometer [30] and the mini Mitter Actical accelerometer [25].

In thirteen (81%) articles authors reported the epoch selected; this was usually 60 seconds [13,16,17,19,22,27,28,30] with individual studies using shorter epochs, namely two [24], five [18], 10 [15], and 15 [14] seconds. Subjects were asked to wear the accelerometer for between two [22] to seven days [13,14,17,18,27,28], with most requesting seven days wear. The criteria used to determine number of days of accelerometer data per child required to characterise habitual activity were specified in 13 articles [13-18,23-25,27-29]: these ranged from one [13,18,29] to four days [17]. In five at least one weekend day was stipulated [14-16,24,30]. In eleven articles duration of wear in any single day was specified [13,15-18,23,24,26-29]: these ranged from at least 360 minutes (six hours) [26] to at least 600 minutes (ten hours) [16-18,23,27-29]. Moderate to vigorous PA (MVPA) was defined using a range of count values varying from 2000 [16] to 3600 [28] cpm. Vigorous PA was defined using Trost *et al.*[32] and a threshold of 1000 [22] cpm.

### Main findings

A seasonal variation in PA levels was reported in all of the UK studies [14,18,24,26-28]. Mattocks *et al.*[27] evaluated seasonal and intra-individual variation of PA in 315 11 year old UK children by obtaining four repeat accelerometer measurements over a full calendar year and calculated intraclass correlation coefficient (ICC) values to determine the extent to which activities in different seasons vary. ICC values for variation in activity (cpm) over the course of the year increased from 0.49 to 0.53 after adjusting for month of measurement, indicating an effect of month. PA levels were higher in summer than winter [27]. Using a cross-sectional study design, Owen *et al.*[18] examined ethnic differences in mean daily PA (cpm) in a cross-sectional study of 144 11 year old UK children, adjusting for month of measurement: PA was higher in summer than winter and did not vary by sex [18]. Riddoch *et al.*[28] compared PA across all four seasons in 5595 11 year old UK children: PA levels (cpm and MVPA) were lowest in winter compared to summer (*n* = 5595).

Wennlöf *et al.*[29] compared the PA levels (cpm) of 969 nine and 15 year old Swedish children who were measured once only in spring, autumn, or winter and found that children were most active during April and May with a significant effect of month of measurement in total PA being observed. Rundle *et al.*[19] compared the activity levels of 437 four year old US children measured either during summer or winter and found season of measurement to be the strongest predictor of mean activity counts with children more active during summer than winter.

By contrast, no variation according to the season of measurement was found in four studies, none of these were carried out in the UK [13,16,22,30], and only one of which compared PA levels (cpm) across all four seasons [30] in 219 US children measured once throughout the year. Bringolf-Isler *et al.*[13] examined the association between PA (cpm) and socio-demographic and environmental characteristics, including season in a longitudinal study of 189 six to 14 year old Swiss children who were measured once in either winter or summer. Nilsson *et al.*[16] determined between- and within- day differences in total PA (cpm), and the daily time spent in MVPA in 1954 nine and 15 year old children from four European countries. They found that adjustment for season of PA measurement (autumn, winter and spring) did not alter their main findings. Finn *et al.*[22] also found no differences between summer and autumn total PA (cpm) and vigorous activity in 214 three to five year old US children.

In only three articles [14,16,26] was seasonal variation in children’s SB evaluated: two from the UK [14,26], and all cross-sectional. Fisher *et al.*[26] compared total PA (cpm) and percentage time spent in SB, light PA, and MVPA across all four seasons in 209 Scottish children (mean age 4.8 years): children were more sedentary in spring than summer. King *et al.*[14] explored 22 potential correlates including season of total PA (cpm), MVPA and SB in 480 seven year old UK children and found that SB was higher in spring, autumn and winter compared to summer. In contrast, Nilsson *et al.*[16] reported that levels of SB in nine and 15 year old European children were not influenced by season of measurement (including only spring, autumn, and winter).

In three articles seasonal PA data were analysed separately for different age groups [15,23,25]. Kristensen *et al.*[23] compared PA levels (cpm) across all four seasons and reported an effect of age: season had less influence on 14 to 16 year olds than in eight to 10 year olds. Overall, European children were more physically active in the spring than in winter or autumn, with the exception of adolescents. Kolle *et al.*[15] compared PA levels (cpm) across spring, autumn, and winter, and found that season had less influence on PA in Norwegian 15 year olds than in Norwegian nine year olds: younger children were more active during spring than during winter and autumn, but the PA levels of 15 year olds did not vary by season. Taylor *et al.*[25] compared total PA (cpm) across all four seasons in New Zealand children at age three, four and five years. Seasonal variation in PA differed by age group: children were less active during spring than during summer or winter at three years, but these differences were not observed at four and five years.

Rowlands *et al.*[24] are the only authors to have considered the influence of gender on seasonal variation of PA. In a longitudinal study of only 64 nine to 11 year old UK children measured in only one of two seasons (summer and winter). Total PA (cpm) and moderate activity were higher during summer than winter for boys on weekend and weekdays, and higher for girls on weekend days. For boys, vigorous activity was higher during summer than winter on weekdays, while weekday activity was higher in summer than winter; for girls, weekend activity was higher in summer than winter. Rowlands *et al.*[24] are also the only authors to have assessed seasonal variation in PA patterns according to the frequency, intensity and duration of PA bouts (lasting greater than four seconds) of light, moderate, and vigorous intensity activity. They found that the mean duration of all intensity PA bouts was greater during summer than winter in boys, and the frequency and intensity of vigorous intensity PA bouts was greater during summer than winter in girls [24]. There are no reports of variation in PA or SB by ethnicity, socioeconomic status, weight status or region of residence.

## Discussion

### Key findings

In summary, seasonal variation in PA was reported in the majority of studies, particularly in children living in the UK, and in younger rather than older children. These seasonal differences were reported in all UK studies (*n* = 6), being highest during summer and lowest during winter. Findings from non-UK studies were inconsistent: seasonal variation in PA was reported in seven studies, but not in four. Findings from non-UK studies that were based in the same country were also inconsistent. For example, one US study reported higher levels of PA in summer than winter [19]; however, a different US study reported higher levels of PA in autumn than summer [22], and one found no association between season and PA levels [30]. Findings were inconclusive for SB and vigorous PA.

### Strengths and limitations of review

This review identified a large number of potential studies obtained from a systematic literature search conducted in a range of databases. The broad definition of search terms and systematic search strategy should have enabled this review to detect as many potential studies as possible. Only studies with accelerometer-determined measurements of activity were included as they are currently regarded as the optimal method for measurement of PA and SB in population studies [33], providing greater accuracy and reliability than self-report methods [34]. This review provides the best summary to date of the evidence evaluating seasonal variation in accelerometer-determined PA and SB in children.

The heterogeneity in study samples, design, accelerometer protocols, statistical methods and outcome measures included in this review limited interpretation and meant that it was not possible to meta-analyse the results. The semi-quantitative reporting in this review provides only a somewhat arbitrary classification of seasonal variation with the focus on specifying seasons characterised by low or high activity rather than the strength of variation. Only one reviewer undertook the reviewing process and no robust indicator of methodological quality was provided due to the lack of uniformity across research methodology. A number of the articles have drawn data from the same cohort studies, for example the Avon Longitudinal Study of Parents and Children [27,28] and the European Youth Heart Study [16,23,29], which may introduce bias into the analysis sample. As this review was restricted to published studies only, publication bias may be present.

### Strengths and limitations of studies

Limitations across the studies included small sample sizes, inconsistent study designs and accelerometer protocols, and the use of varied seasonal definitions and statistical methods. There were also few studies that employed a repeat measures study design, the most appropriate design allowing differentiation between seasons and between subjects. The studies included in the current review comprised geographically clustered samples, limiting the representativeness of their findings. Few studies evaluated seasonal variation in SB or in vigorous PA independently of total PA. There were also limited and conflicting evidence regarding sex and age effects on seasonal variation in PA therefore no conclusions can be reached on this.

However, there were a sufficient amount of UK studies conducted in a range of settings that reported consistent findings. In addition, only studies using accelerometers were included in this review because they provide more accurate estimates of volumes and intensities of PA than self-report measures [35]. However, there are limitations associated with the use of accelerometers including underreporting levels of cycling, and their inability to accurately measure swimming or load bearing activity. Despite recent advances in accelerometer design allowing researchers to select shorter epochs whilst still being able to measure for a sufficient number of days, a 60 second epoch was used in half the studies included in this review. Due to the sporadic nature of children’s PA, longer epochs may be inappropriate when measuring PA and may underestimate levels of MVPA [36]. It has also been widely documented that the use of different thresholds to define activity intensities limits the ability for researchers to make reliable comparisons of MVPA levels between studies, and at present there is still no consensus on the best threshold to use [37].

### Further research needed

Future studies of seasonal activity in children need to use large samples, employ a repeat measures study design, use comparable definitions of season, and use a consistent standardised approach to accelerometer measurement. This should include the use of a short epoch, presenting count data as well as minutes of MVPA, and also take into account activities that cannot be measured accurately by accelerometers.

Further research is also needed to understand seasonal variation in children’s SB and vigorous PA independently of total PA so that public health interventions aimed at reducing SB and increasing vigorous PA can be targeted at specific times of the year. Recent evidence suggests that SB in children is associated with an elevated metabolic risk profile [38,39], elevated blood pressure [40], and increased body weight [41-46], independent of PA. Furthermore, participation in vigorous intensity PA appears more consistently associated with lower adiposity [47-49] than total PA [47,50], and this may even be independent of time spent in SB [43].

Seasonal variation in PA appears to be location specific; therefore additional research is needed in different countries and within different regions of larger countries. Characteristics that define seasons including the weather, ecology, and hours of daylight vary according to country, and even within different regions of large individual countries. For example, winter in the UK is different to winter in Norway in terms of weather and also available sunlight, and the weather in the US state of New York (Rundle *et al.*[19]) is characterised by cold, snowy winters and usually warm summers, whereas South Dakota (Finn *et al.*[22]) usually experiences cold winters and hot summers that can bring thunderstorms with high winds, thunder and hail.

Regional differences in seasonal variation may reflect variations in climate. As the meteorological factors associated with seasons in a specific region cannot be altered, there may be a role for future research to study other factors associated with variation of PA and SB throughout the seasons. This will enable us to understand what encourages a child to be more or less physically active in specific seasons. For example, previous research [51] evaluating regional differences in pedometer-determined PA between urban and rural primary children living in Cyprus found an interaction between season and rural/urban regions: rural children were more active in summer, and urban children were more active in winter. To our knowledge, the only study evaluating seasonal differences in accelerometer-determined PA in rural and urban children is reported in a published abstract by Tremblay *et al.*[52]. The authors found that rural children were more active during summer and urban children more active in the winter.

Additional research is needed that examines possible interactions of season with other factors known to influence PA and SB such as sex, age, ethnic group, weight status and geographic location [53-56]. In contrast to Carson *et al.*[9]*,* we found some evidence to suggest that season influenced children but not adolescents. This may be because adolescents have less free time to play outdoors than children; therefore, their PA is less influenced by fluctuations in daylight and weather. Adolescents are also more likely to participate in organised PA that takes place all year around [57]. Sex differences in PA across season may relate to the type of playground activities typically undertaken by boys and girls at school [24]. Boys undertake more vigorous activity during play time at school than girls and therefore warm summer weather may have more impact in the amount of PA that they undertake [57].

In the majority of studies identified for this review, authors reported a measurement of weight and several reported the ethnic composition of their sample; however, none determined whether seasonal variation in activity were associated with weight status’, or ethnic group. Overweight children are less likely to be active than underweight children [58,59], and British South Asian children are less likely to be active than European whites and black African-Caribbeans [18,60-63]. It is possible that the influence of season on PA and SB may be enhanced in specific ethnic groups, or in overweight children compared to underweight children. It is important to identify factors that may cause or interact with seasonal effects in PA and SB, so that interventions can target certain groups of children.

## Conclusion

From our review we suggest there is sufficient evidence to develop public health interventions to increase PA during winter in UK children. There is insufficient high quality evidence to suggest this for children living in other countries and this may reflect regional variations in climate as well as varying definitions of season. No conclusions can be drawn regarding the effect of season on children’s SB or vigorous PA reflecting few studies of small sample size, lack of repeat measures, incomparable definitions of season and inconsistent accelerometer protocols. More research is needed to determine whether levels and patterns of vigorous PA and SB vary according to season utilizing a consistent standardised approach to accelerometer measurement, a repeated measures study design and in large geographically dispersed samples. Future research should be aimed at addressing the gaps in evidence identified in this review namely seasonal effects on SB and vigorous PA, and the determinants of seasonal patterns in PA such as age, sex, and geographic and climatic setting so that interventions can be season-specific and target inactive children.

## Competing interests

The authors declared that they have no competing interest.

## Authors’ contributions

CR was responsible for the conception and design of the review, the literature search, critical appraisal of the included papers, and also drafted the manuscript. LJG and CD made substantial contributions towards the conception and design of the review and revising the manuscript. All authors read and approved the final manuscript.

## Supplementary Material

Additional file 1 **Literature search strategy.** Literature search strategy used in electronic databases. Click here for file

Additional file 2 **Table S1.** Articles (*n* = 16) evaluating the influence of season on accelerometer-determined PA and SB in children. Click here for file
