# Supplementary material for: Seasonal variation in accelerometer-determined sedentary behaviour and physical activity in children: a review
Source: Int J Behav Nutr Phys Act. 2012 Apr 30;9:49. doi: 10.1186/1479-5868-9-49 (PMC3511197; doi:10.1186/1479-5868-9-49)
Supplement: Additional file 1 — Literature search strategy. Literature search strategy used in electronic databases. [file 1479-5868-9-49-S1.doc]

Table 1: Literature search strategy

| **Search term number** | **Search term** | **No. of search hits** |
| --- | --- | --- |
| 1 | explode “Child, Preschool” / all SUBHEADINGS in MIME, MJME | 655389 |
| 2 | explode “Child” / all SUBHEADINGS in MIME, MJME | 1355604 |
| 3 | explode “Adolescent” / all SUBHEADINGS in MIME, MJME | 1372756 |
| 4 | explode “Motor- Activity” / all SUBHEADINGS in MIME, MJME | 91462 |
| 5 | explode “Energy Metabolism” / all SUBHEADINGS in MIME, MJME | 237169 |
| 6 | explode “Sedentary Lifestyle” / all SUBHEADINGS in MIME, MJME | 420 |
| 7 | explode “Television” / all SUBHEADINGS in MIME, MJME | 24918 |
| 8 | ((physical or motor) activity) or energy or sedentar* or television or computer* | 1073263 |
| 9 | acceleromet* | 3723 |
| 10 | (explode “Child, Preschool” / all SUBHEADINGS in MIME, MJME) or (explode “Child” / all SUBHEADINGS in MIME, MJME) or (explode “Adolescent” / all SUBHEADINGS in MIME, MJME) | 343601 |
| 11 | (explode “Motor- Activity” / all SUBHEADINGS in MIME, MJME) or (explode “Energy Metabolism” / all SUBHEADINGS in MIME, MJME) or (explode “Sedentary Lifestyle” / all SUBHEADINGS in MIME, MJME) or (explode “Television” / all SUBHEADINGS in MIME, MJME) or ((physical or motor) activity) or energy or sedentar* or television or computer* | 1263011l |
| 12 | explode “Seasons” / all SUBHEADINGS in MIME, MJME | 63066 |
| 13 | explode “Weather” / all SUBHEADINGS in MIME, MJME | 357786 |
| 14 | variation or variab* or wind or humid* or rain* or snow* or sun* or temperat* or cold or hot or months or spring or summer or autumn or fall or winter | 2259441 |
| 15 | (explode “Seasons” / all SUBHEADINGS in MIME, MJME) or (explode “Weather” / all SUBHEADINGS in MIME, MJME) variation or variab* or wind or humid* or rain* or snow* or sun* or temperat* or cold or hot or months or spring or summer or autumn or fall or winter | 2347299 |
| Final search | 9 and 10 and 11 and 25 | 219 |
