# Supplementary material for: Seasonal variation in accelerometer-determined sedentary behaviour and physical activity in children: a review
Source: Int J Behav Nutr Phys Act. 2012 Apr 30;9:49. doi: 10.1186/1479-5868-9-49 (PMC3511197; doi:10.1186/1479-5868-9-49)
Supplement: Additional file 2 — Table S1. Articles (n = 16) evaluating the influence of season on accelerometer-determined PA and SB in children. [file 1479-5868-9-49-S2.doc]

**Table 1 Articles (*n* =**16) evaluating the influence of season on accelerometer-determined PA and SB in children

| **Authors; study name (if applicable)** | **Year; country, region** | **Method of recruitment** | **Study consent rate (%)** | **Sample [Size; gender; mean age (±SD)]** | **Period of data collection** | **Description of study** | **Accelerometer protocol (model; no. days wear; epoch; valid data; intensity cut points)** | **Valid accelerometer data receipt (%)** | **Season definition** | **Statistical methods** | **Main PA results** | **Main SB results** | **Finding of seasonal variation in PA** | **Finding of seasonal variation in SB** |
| --- | --- | --- | --- | --- | --- | --- | --- | --- | --- | --- | --- | --- | --- | --- |
| Bringolf-Isler *et al* [13]; The Swiss surveillance program on childhood allergy and respiratory symptoms | 2009; Switzerland, Bern, Biel-Bienne and Payerne | Study | 80.0 | *n=*189; 81m, 88f; 6-7y, 9-10y & 13-14y (range) | Win 2004 & Spr/ Sum 2005 | Within subject repeat measures in 2 seasons | Actigraph 7164; 7d wear; 60 sec epoch; Valid data:≥1d, ≥480min/d | 86.0 | Win: NS; Sum: NS | Mixed linear regression | No difference in PA(cpm) between win & sum (638 vs. 670 cpm; *p*>0.05) | NS | No | NS |
| Burdette *et al* [30] | 2004; US, Ohio | Advertising | NS | *Total n*=214; *Spring sample:* *n*=28, *Summer sample:* *n*=39, *Autumn sample:* *n*=89, *Winter sample:* *n*=58; 122m, 92f; 44 (range 29-52)mth | Jul 2000- Jun 2001 | Between subject measures in 4 seasons | Tritac R3D; 3d wear (inc. 2w/d & 1dw/e); 60 sec epoch; Valid data:NS | NS | Spr: Mar- May; Sum: Jun- Aug; Aut: Sep- Nov; Win: Dec- Feb | NS | No difference in PA (cpm) across 4 seasons (*p*>0.05); win PA tend to be lower (*p*=0.05) | NS | No | NS |
| Finn *et al* [22] | 2002; USA, South Dakota | Childcare centre | NS | *n*=214; 106m, 108f; 3.93 (0.06)y | NS | Between subject measures in 2 seasons | Actiwatch AW16; 2d wear; 60 sec epoch; Valid data:NS; Vigorous:1000cpm | 90.0 | Sum: NS; Aut: NS | Forward-backward stepwise regression | No difference in total daily counts (*p*=0.28) or % time spent in vigorous PA (*p*=0.35) between sum & aut; higher PA in aut compared sum between 09:00 and 19:00 (159.9 vs. 131.2 cpm; *p*<0.05) | NS | Yes | NS |
| Fisher *et al* [26]; Study of Preschool Activity, Lifestyle and Energetics | 2005; Scotland, Glasgow | Study | NS | *Total n*=209; 101m, 108f; *Spring sample*: *n*=70; 40m, 30f; *Summer sample:* *n*=52; 24m, 28f; *Autumn sample: n*=41; 13m, 28f; *Winter sample:* *n*=46; 25m, 21f; 4.8 (1.2)y | Feb 2001- Jul 2002 | Between subject measures in 4 seasons | Actigraph 7164; 3d wear (younger children), 7d wear (older children); Epoch: NS; Valid day: 360min/d; SB:<1100cpm, light:1100-3200cpm, MVPA: >3200cpm | 97.0 | Spr: Feb- Apr; Sum: May- Jul; Aut: Aug- Oct; Win: Nov- Jan | ANOVA | *Total PA:* Lower PA in spr than sum (mean diff =125 cpm; *p*<0.001), aut (mean diff=118 cpm; *p*<0.01), & win (mean diff=77 cpm; *p*<0.05), *Light PA:* Lower light PA (*p*<0.05) in spr than sum (mean diff=4.3% of monitored time) or aut (mean diff=2.8 % of monitored time) *MVPA:* Lower MVPA (*p*>0.05) in spr than sum (mean diff=4.3% of monitored time) & aut (mean diff=1.0% of monitored time) | Seasonal differences in % time spent SB (*p*<0.001); Higher (*p*<0.05) in spr than sum (mean diff=4.8% of monitored time) or aut (mean diff=2.9% of monitored time) | Yes | Yes |
| King *et al* [14]; Gateshead Millennium Study | 2010; England, Tyne and Wear | Study | 83.5 | *n*=480; 244m; 236f; 7y | 2006- 2007 | Between subject measures in 4 seasons | Actigraph 7164 & GT1M; 7d wear; 15 sec epoch; Valid number days:≥2d & 1d w/e; SB:<1100cpm, MVPA:≥3200cpm | 94.7 | Spr/ Aut: Mar- Oct; Sum: May- Aug; Win: Jan- Dec | Linear regression | PA (cpm) lower in win, & spr/aut than sum (β coefficient: 0.0033 to 0.058; *p*<0.001); MVPA lower in spr/ aut than sum (β coefficient: 0.087 to 0.208; *p*<0.001) | SB higher in spr/ aut & win than summer (-2.907 to -1.684; *p*<0.001). | Yes | Yes |
| Kolle *et al* [15] | 2009; Norway, all regions | School | NS | *Total n*=1824; 940m, 884f; *Age 9y sample*: *n*=1127; 602m, 525f; *Age 15y sample:* *n*=697; 338m, 359f | Mar 2005- Oct 2006 | Between subject measures in 3 seasons | Actigraph 7164; 4d wear (inc. 1d w/e); 10 sec epoch; Valid data:≥2d, ≥480min/d | 79.0 | Spr: 1 Mar- 15 June; Aut: 1 Sep- 30 Nov; Win: 1 Dec- 28 Feb | Linear regression | *Age 9y:* Higher in spr than win (diff girls: 188 cpm; CI: 112 to 265; *p*<0.001; diff boys: 121 cpm; 41 to 201; *p*=0.001) & aut (girls diff: 112 cpm; CI: 57 to 167; *p*<0.001; boys diff: 113 cpm; CI: 53 to 172; *p*<0.001)  *Age 15y:* No differences in cpm between seasons (*p*>0.05) | NS | *Age 9y:* Yes; *Age 15y:* No | NS |
| Kristensen *et al* [23]; European Youth Heart Study | 2008; Denmark, Odense | Study | *1997 sample Children:* 76.0; Adolescents: 65.0 *2003 sample Children: 57.6; Adolescents: 65.0* | *1997 sample Children: n*=377; 177m, 200f; 9.7 (0.4)y; Adolescents: *n*=232; 102m, 130f; 15.5 (0.5)y; *2003 sample: Children: n*=416; 179m, 237f; 9.8 (0.4)y; Adolescents: *n*=305; 134m, 171f; 15.8 (0.4)y; | 1997 & 2003 | Between subject comparisons in 4 seasons in 2 groups of children (1997 & 2003) | Actigraph 7164; 5d wear; Epoch:NS; Valid data:≥3d, ≥600min/d | 69.3 | Spr: Mar- May; Sum: Jun- Aug; Aut: Sep- Nov; Win: Dec- Feb | Linear regression | *Age 8-10y:* Highest PA (cpm) in spr than win & aut months in 1997 & 2003 (*p*<0.0001)  *Age 14-16y:* No diff in PA(cpm) between months in 1997 & 2003 (*p*=0.06) | NS | *Age 8-10y:* Yes; *Age 14-16y:* No | NS |
| Mattocks *et al* [27]; Avon Longitudinal Study of Parents and Children | 2007; England, Avon | Study | 64.0 | *n*=315; 148m, 167f; 11.65 (0.19)y | NS | Within subject repeat measures in 4 seasons | Actigraph 7164; 7d wear; 60 sec epoch; Valid data:≥3d, ≥600min/d; | *Season 1:* 90.0; *Season 2:* 86.0; *Season 3:* 81.0; *Season 4:* 78.0; *All 4 seasons:* 70.0 | Spr: Feb- Apr; Sum: May- Jul; Aut: Aug- Oct; Win: Nov- Jan | ICC values | PA (cpm) highest sum & lowest win (diff: 93 cpm; *p*: NS) | NS | Yes | NS |
| Nilsson *et al* [16]; European Youth Heart Study | 2009; Denmark (Odense), Portugal (Madeira) , Estonia (Tartu) & Norway (Oslo) | Study | NS | *n*=1954; 918m, 1037f; 9.7 (0.4)y & 15.5 (0.5)y | 1997 & 2003 | Between subject comparisons in 3 seasons in 2 groups of children (1997 & 2003) | Actigraph 7164; 4d wear (inc. 2 w/e); 60 sec epoch; Valid data:≥3d (inc. 1 w/d), ≥600min/d; SB:<100cpm, MVPA:≥2000cpm | NS | Spr: NS; Aut: NS; Win: NS | Repeat measures ANOVA (adjusted for season) | Adjusting for the season of measurement did not change PA (cpm & MVPA) | Adjusting for season of measurement did not change SB | No | No |
| Nyberg *et al* [17]; Stockholm Obesity Prevention Project | 2007; Sweden, Stockholm | School | NS | *n*=97; 41m, 56f; *Baseline age:* 7.5 (0.92)y; *Follow-up age:*  9 (0.92)y | 2002- 2005 | Within subject repeat measures in 2 periods between 2002 - 2005 | Actiwatch-4; 7d wear; 60 sec epoch; Valid data:≥4d , ≥600min/d | 92.0 | Dark: Oct- Mar; Light: Apr- Sept | Linear regression | Difference in PA (cpm) higher in dark followed by light measurements & light followed by light measurements than light followed by dark measurements (*p*=0.001& *p*=0.024, respectively) | NS | Yes | NS |
| Owen *et al* [18]; Child Heart and Health Study | 2009; England, London, Birmingham and Leicester | Study | 69.0 | *n=*2144; 1029m, 1115f; 9.9 (range 9.2-10.7)y | Jan 2006- Feb 2007 | Between subject comparisons across 4 seasons | Actigraph GT1M; 7d wear; 5 sec epoch; Valid data:≥1d, ≥600min/d | 60.0 | Monthly | Multilevel linear regression | PA (mean activity counts & cpm) higher in sum months than win months | NS | Yes | NS |
| Riddoch *et al* [28]; Avon Longitudinal Study of Parents and Children | 2007; England, Avon | Study | 93.0 | *n*=5595; 2262m; 2933f 11.79 (SD 0.24)y | Jan 2003- Jan 2005 | Between subject comparisons across 4 seasons | Actigraph 7164; 7d wear; 60 sec epoch; Valid data:≥3d, ≥600min/d; MVPA: ≥3600cpm | 78.2 | Spr: 1 Mar- 31 May; Sum: 1 Jun- 31 Aug; Aut: 1 Sep– 30 Nov; Win: 1 Dec- 28 Feb | ANOVA | PA (cpm & MVPA) were highest in sum & lowest in win (*p*<0.001) | NS | Yes | NS |
| Rowlands *et al* [24] | 2009; England, southwest | School | NS | *n*=64; 32m, 32f; 9.9 (0.3)y | Jan/ Feb 2007 & Jun/ Jul 2007 | Within subject repeat measures in 2 seasons | Actigraph GT1M; 6d wear; 2 sec epoch; Valid data:≥3d (w/d), ≥600min & ≥1d (w/d); ≥ 480min; Moderate & vigorous:Trost *et al* [32] | 76.2 | Sum: Jun- Jul; Win: Jan- Feb | Repeated measure ANOVA; ICC values | *Total PA:* PA (cpm & moderate PA) higher in sum than win for boys on w/e & w/d & higher for girls on w/e (*p*<0.05); PA (vigorous PA) higher in sum than win for boys on w/d (*p*<0.05) *PA bouts:* PA bout duration (light, moderate & vigorous) higher in sum than win (*p*<0.05) in boys; PA bout frequency & intensity (vigorous) higher in sum than win (*p*<0.05) in girls *Consistency of PA:* PA less seasonal variation in girls than boys when including w/d & w/e (girls ICC: 0.72- 0.90; boys ICC: 0.63- 0.68; all *p*<0.01) | NS | Yes | NS |
| Rundle *et al* [19]; New York City Head Start | 2009; USA, New York | Study | NS | *n*=437; 213m, 224f; 50.7 (8.0)m | Jun 2003- Jan 2006 | Between subject comparisons in 2 seasons | Actiwatch; 6d wear; 60 sec epoch; Valid data:NS | 84.6 | Sum: May- Sep; Win: Oct- Apr | Linear regression | PA (cpm) higher in sum than win (β coefficient: 87.04; *p*<0.001) | NS | Yes | NS |
| Taylor *et al* [25]; Family Lifestyle, Activity, Movement and Eating Study | 2008; New Zealand, Dunedin | Study | 59.0 | *Total n*=574; 319m, 255f; *Age 3y sample:* *n*=208; 113m, 95f; *Age 4y sample:* *n*=180; 101m, 79f; *Age 5y sample:* *n*=186; 105m, 81f; | 2004- 2007 | Longitudinal between subject measures between 3 seasons at 3 different ages | Mini Mitter Actical; 5d wear; Epoch: NS; Valid data:≥3d, length of day calculated from parental report; | *Age 3y sample:* 93.0; *Age 4y sample:* 86.0;  *Age 5y sample:* 96.0 | Spr: Sep- Oct; Sum: Nov- Feb; Aut: Mar- May; Win: Jun- Aug | Linear regression | *Age 3y:* PA (cpm) lower in spr than sum or win (*p*<0.001) *Age 4 & 5y:* No difference in PA (cpm) across 3 seasons at 4y & 5y (*p*=0.974 & *p*=0.383, respectively) | NS | *Age 3y:* Yes; *Age 4 & 5y:* No | NS |
| Wennlöf *et al* [29]; European Youth Heart Study | 2005; Sweden, Örebro | Study | 50.0 | *Total n*=969; 471m, 498f; *Age 9y sample*: 9.5 (range 8.5- 10.3)y; *Age 15y sample:* 15.6 (range 14.7- 16.4)y | Spr 1999 | Between subject comparisons in 3 seasons | Actigraph 7164; 3-4d wear; Epoch:NS; Valid data:≥1d; ≥600min/d; | 69.0 | Spr: Mar- May; Aut: Sep- Nov; Win: Dec- Feb | Three way ANOVA (age, sex, season) | PA (cpm) highest during Apr & May; Significant effect from month of measurement in cpm (*p*<0.001) | NS | Yes | NS |

Abbreviations: *n* = sample size; m = males; f = females; y = years; mth = months; spr = spring; sum = summer; aut = autumn; win = winter; NS = not specified; Jan = January; Feb = February; Mar = March; Apr = April; Jun = June; Jul = July; Aug = August; Sep = September; Oct = October; Nov = November; Dec = December; d = days; min = min; sec = seconds; cpm = counts per min; SB = sedentary behaviour; MVPA = moderate and vigorous PA; w/d = weekday; w/e = weekend; PA = PA; RSK = rural Saskatchewan; OOM = Old Order Mennonite
